# Adenocarcinoma of the cervix involving the fallopian tube mucosa: report of a case

**DOI:** 10.1186/s13000-016-0529-8

**Published:** 2016-08-17

**Authors:** Chelsey D. Deel, Richard A. Allen, Laura L. Holman, Rosemary E. Zuna

**Affiliations:** 1 Department of Pathology, University of Oklahoma Health Sciences Center, Oklahoma City, OK 73104 USA; 2 Division of Gynecologic Pathology, Department of Obstetrics and Gynecology, University of Oklahoma Health Sciences Center, Oklahoma City, OK 73104 USA

**Keywords:** Fallopian tube, Adenocarcinoma, Cervix, Case report

## Abstract

**Background:**

Fallopian tube involvement by cervical carcinoma has rarely been documented, with literature reports focusing primarily on squamous cell carcinoma.

**Case presentation:**

In this report, we present the case of a 50 year old woman who presented with an abnormal Pap test with atypical squamous and glandular cells. A loop electrosurgical excision procedure (LEEP) was performed and led to the diagnosis of stage IB1 endocervical adenocarcinoma. Subsequent radical hysterectomy, bilateral salpingo-oophorectomy, and bilateral pelvic lymph node dissection showed a well-differentiated endocervical adenocarcinoma of usual type with superficial spread to the endometrium and right fallopian tube. The patient received no adjuvant therapy and has remained without evidence of disease.

**Conclusions:**

While the advent of more extensive fallopian tube sampling has led to increased discovery and discussion of fallopian tube involvement by metastatic carcinoma, its impact on treatment and prognosis remains to be seen.

## Background

Because fallopian tube carcinoma was thought to be a rare occurrence, the fallopian tube had not been emphasized in gynecologic malignances until rather recently. The recognition that serous carcinomas of ovarian type frequently involved the fallopian tube fimbria [1] has recently led to the concept that, in fact, the fallopian tube epithelium was the origin of most high grade ovarian serous carcinomas. There is now a strong interest in establishing the role of the fallopian tube in gynecologic malignancies and, increasingly, the entire fallopian tube is being submitted for histologic analysis.

In this report, we present a case of a 50 year old woman who was diagnosed with stage IB1 well-differentiated endocervical adenocarcinoma with surface extension to the endometrium and fallopian tube, confirmed by histologic and immunohistochemical analysis. Fallopian tube involvement by cervical adenocarcinoma is a rarely reported entity with as yet unknown effects on patient outcome. The introduction of more extensive adnexal sampling may yield similar cases and encourage further discussion of its significance to treatment and survival.

## Case presentation

A 50 year old woman with a normal Pap smear history presented in 2012 with an abnormal Pap smear that showed atypical squamous cells, cannot exclude high grade lesion (ASC-H) and atypical glandular cells. Cervical biopsy and endocervical curettage (ECC) showed atypical glandular/endocervical epithelium. A loop electrosurgical excision procedure (LEEP) was performed, revealing well-differentiated adenocarcinoma of the cervix with mucinous and intestinal features. The tumor measured 12 mm in horizontal spread and invaded 8 mm into the stroma. The deep stromal, endocervical, and ectocervical margins were positive. PET/CT was negative for metastatic disease. Given her apparent stage IB1 disease, the patient was dispositioned to radical hysterectomy, bilateral salpingo-oophorectomy, and bilateral pelvic lymph node dissection.

**Fig. 1:**
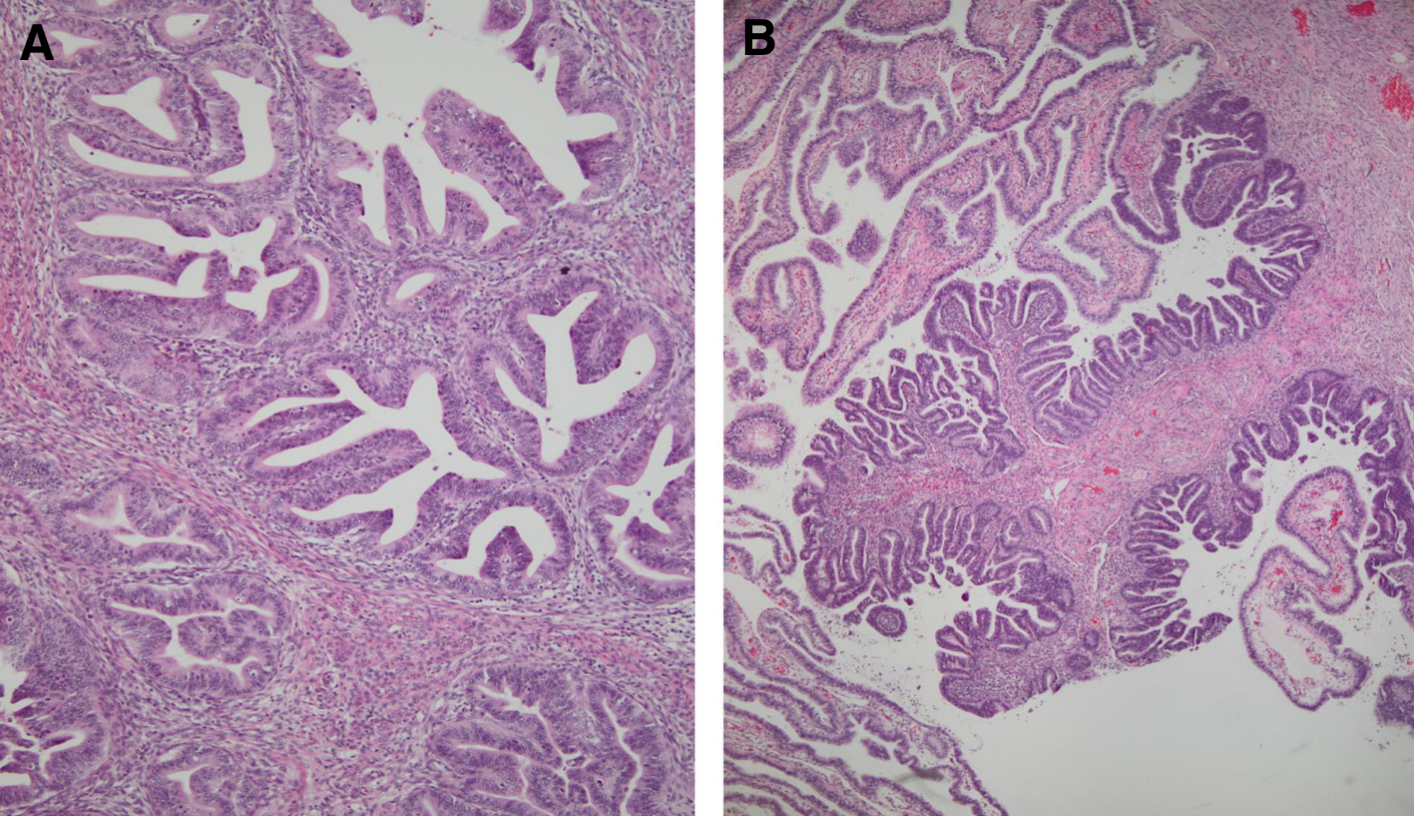
Histopathology of cervical adenocarcinoma. **a** The primary cervical tumor shows well differentiated, mucin depleted glands (Hematoxylin and eosin, 200x original magnification); **b** Identical tumor tissue involves the fallopian tube mucosa. There is a sharp demarcation between the tumor and the normal tubal mucosa. (Hematoxylin and eosin, 200x, original magnification)

**Fig. 2:**
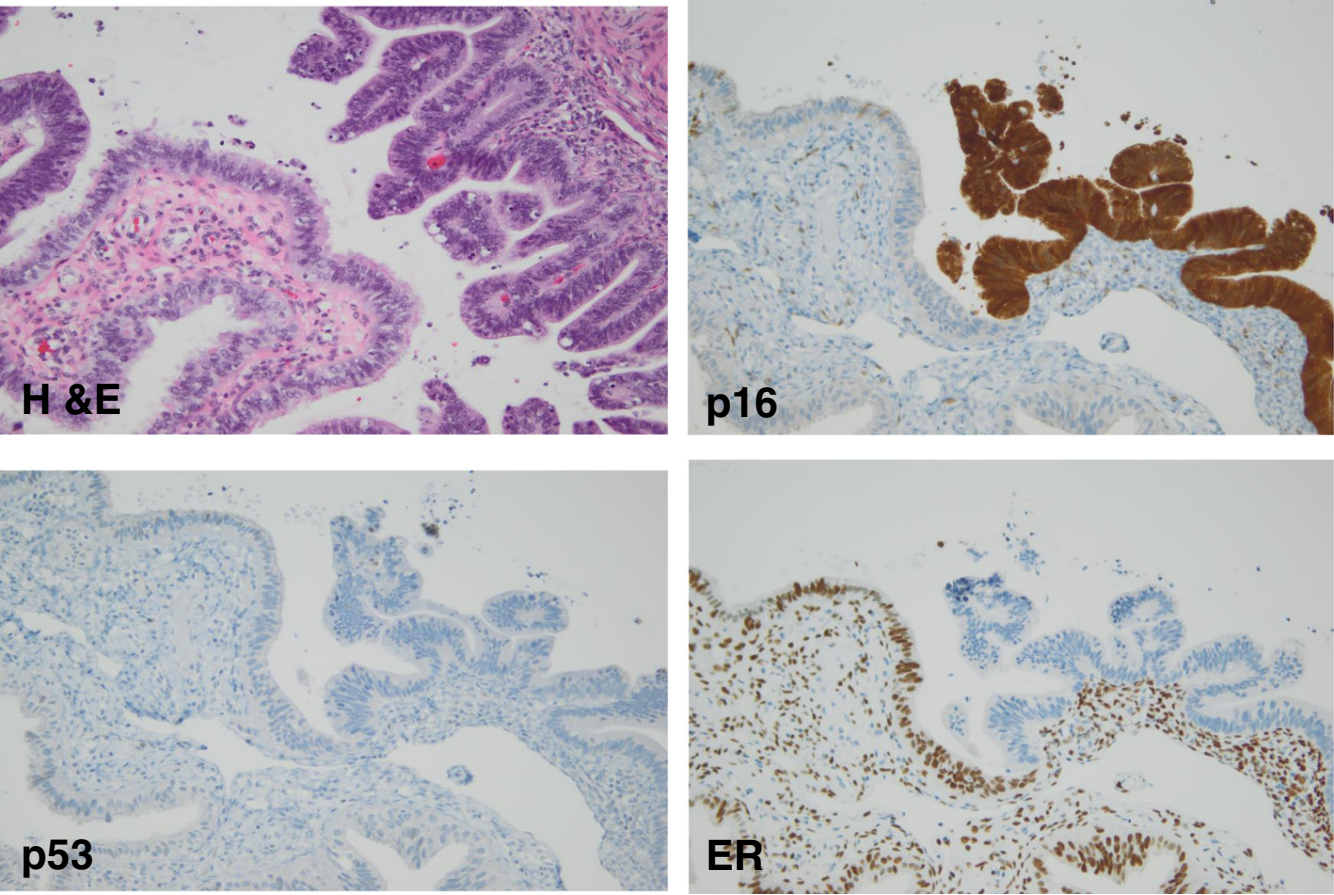
Staining pattern of the tubal metastasis. The higher power image of the tubal lesion, hematoxylin and eosin (400x, original magnification). Immunohistochemical stains of the tubal tumor show the contrast with the normal tubal tissue. p16 is strongly diffusely positive in the tumor unlike the normal mucosa (400x, original magnification); p53 shows a wild type pattern similar to the normal mucosa (400x, original magnification); Estrogen receptor (ER) is negative in the tumor tissue in contrast to the normal (400x, original magnification). This pattern contrasts with the expected pattern for high grade serous carcinoma arising in the fallopian tube which would typically show positive staining for ER and p53 (mutated)

Macroscopically, the cervix revealed a red-tan granular lesion measuring 3.5 × 3.5 cm. The uterine cavity was lined with a red-tan, shaggy endometrium measuring up to 0.4 cm in thickness. The bilateral fallopian tubes and ovaries appeared grossly normal. Histologically, sections of the cervix (Fig. 1a) revealed a well-differentiated, neoplastic proliferation of glands with focal mucinous and intestinal differentiation. The lesion invaded 7 mm where the wall measured 15 mm with surface extension to the upper genital tract. This included the endometrium and focal non-invasive involvement of the right fallopian tube (Fig. 1b). No lymphovascular space invasion was identified. The parametria, vaginal cuff margins, ovaries, and 26 regional lymph nodes were negative for malignancy. On immunostaining, the tumor cells from the cervix were diffusely positive for CK7 and p16, focally positive for mucicarmine, and negative for ER, CK20, vimentin, CDX2, and p53. An identical staining pattern was found in sections of the endometrium and fallopian tube (Fig. 2), supporting extension of the cervical primary tumor. Formalin-fixed, paraffin-embedded tissues from the cervix and endometrium were genotyped using Roche Linear Array® [2]. Both tissues harbored HPV18 DNA. The morphology and immunostaining pattern supported the diagnosis of invasive adenocarcinoma, endocervical type with focal mucinous and intestinal differentiation, with involvement of the superficial endometrium and right fallopian tube. Given the unknown significance of the tumor in the endometrium and fallopian tube in a patient with otherwise low-risk disease, no adjuvant therapy was recommended. She has remained without evidence of disease since that time (48 mos).

## Conclusions

Cervical cancer remains the third most common gynecologic malignancy in the United States, occupying 0.8 % of all new cancer cases in the U.S. [3] Based on 2008–2012 cases and deaths reported in the SEER registry, the number of new cases of cervical cancer was 7.7 per 100,000 women per year [3]. Importantly, the number of new cases of cervical cancer has been declining steadily over the past decade, primarily due to improved Pap smear screening programs. However, this important trend does not extend to all histologic subtypes of cervical cancer. The rate of squamous cell carcinoma has declined steadily with the advent of Pap testing, but the incidence of adenocarcinoma and adenocarcinoma in situ (AIS) appears to be increasing [4, 5]. Particularly, adenocarcinoma appears to be occurring at an increased frequency in younger women [4, 5]. Several factors have been suggested to explain this trend, including the difficulty in diagnosing glandular lesions via Pap smear and other etiologic factors such as nulliparity, obesity, and changes in oral contraceptive use [3].

Upper genital tract involvement by cervical carcinoma has received little attention, with limited cases reported in the literature [6, 7]. Ovarian metastasis by cervical cancer is well recognized; the incidence is reportedly higher in adenocarcinoma compared to squamous cell carcinoma [8]. The incidence ranges from 2 to 28.6 % in cervical adenocarcinoma and 0 to 17.4 % for squamous cell carcinoma, depending on disease stage [9]. The suggested variable of tumor size greater than 30 mm affects the incidence of ovarian metastasis in cervical adenocarcinoma, while ovarian metastasis in cervical squamous cell carcinoma appears to depend more on clinical stage [8]. Whether due to changing thoughts about the pathogenesis of ovarian cancer or other factors, increased fallopian tube sampling has led to the interesting discovery of metastatic cancers. Rabban et al. [10] reported the pattern and topography of 100 non-gynecologic cancers that metastasized to the fallopian tubes. Most tumors were adenocarcinoma, primarily of colon and breast origin. The metastatic tumors primarily favored the fimbriae of the fallopian tube, with patterns of mucosal growth ranging from flat lesions to lesions with varying degrees of exophytic growth and stratification [10]. These metastatic tumors can mimic primary benign and malignant neoplasms of the fallopian tube, creating a potential diagnostic pitfall [10, 11]. In many cases, a high morphologic index of suspicion along with confirmatory ancillary HPV testing helped make the correct diagnosis [11].

Compared with ovarian metastasis, reports of cervical metastasis to the fallopian tube are extremely rare, with individual case reports primarily of the squamous lesions [6]. Recently, Reyes et al. [9] analyzed 20 cases of cervical carcinoma with secondary involvement of the uterine corpus and adnexa. Fallopian tube metastasis by endocervical adenocarcinoma was seen in 8 of 10 cases, primarily occurring as microscopic lesions [9]. These microscopic lesions showed mucosal colonization mimicking a primary tubal process. To avoid this potential diagnostic pitfall, the authors emphasized the importance of analyzing distinct morphologic as well as immunohistochemical features to differentiate between endocervical adenocarcinoma and primary tubal malignancies.

Histologic evaluation of these metastatic lesions reveal elongated, hyperchromatic nuclei with focal mucinous areas and typical histologic features of cervical adenocarcinoma, including prominent apical mitoses and numerous apoptotic bodies [9]. Immunohistochemistry and in-situ hybridization (ISH) can provide further clarification of the primary source of malignancy. In particular, endocervical adenocarcinomas are characterized by positive HPV ISH and negative p53 overexpression, while serous tubal intraepithelial carcinomas (STIC) exhibit p53 overexpression or null pattern and negative HPV ISH. WT-1 positive immunostaining would also favor a tubal serous neoplasm [12]. Importantly, immunohistochemistry for p16 is not helpful in this differential, as both high-grade serous tumors and cervical adenocarcinomas are typically strongly and diffusely positive. Fallopian tube involvement by metastatic carcinoma, whether gynecologic or non-gynecologic, has received increased attention with the advent of more extensive fallopian tube sampling. The current paradigm of high-grade serous carcinomas of the ovary potentially arising from the fallopian tube may explain this increased sampling phenomenon [10, 13, 14]. The authors suggest that, due to increased sampling, recognition of metastatic fallopian tube involvement by carcinoma may become more frequent.

In our case, morphologic and immunohistochemical patterns confirmed the spread of primary cervical adenocarcinoma to the endometrium and fallopian tube. The proposed mechanism of upper genital tract involvement by cervical carcinoma is contiguous and transtubal spread [6]. The effect of fallopian tube involvement on prognosis and treatment remains unclear. Certainly, there is concern for possible exposure of the tumor to the peritoneal cavity. FIGO staging of cervical carcinoma remains clinical staging and fallopian tube involvement is not mentioned [15]. The present case suggests that the spread of endocervical adenocarcinoma to the endometrium and adnexa may not necessarily be associated with a worse prognosis in patients with disease that is otherwise localized to the cervix. However, additional follow-up studies are needed. In conclusion, upper genital tract involvement by cervical adenocarcinoma remains a rare event, although more extensive adnexal sampling will likely yield additional examples. The impact of tubal involvement by cervical cancer on patient outcome, if any, remains to be established.

## Abbreviations

ECC, endocervical curettings; ER, estrogen receptor; ISH, in situ hybridization; LEEP, loop electrosurgical excision procedure; WT-1, Wilms tumor antigen
